# Case Report: A case of primary IgG4-related skin disease presenting initially with skin papules and abscesses

**DOI:** 10.3389/fimmu.2026.1850129

**Published:** 2026-06-30

**Authors:** Xing-Yue Chen, Jun Chen, Lei Lv, Da-Wei Jiang, Kun-Lan Long, Li-Jia Zhi

**Affiliations:** Department of Critical Care Medicine, Hospital of Chengdu University of Traditional Chinese Medicine, Chengdu, China

**Keywords:** cutaneous manifestation, IgG4-positive plasma cells, IgG4-related disease, IgG4-related skin disease, primary IgG4-related skin disease

## Abstract

**Background:**

Immunoglobulin G (IgG) type 4-related disease (IgG4-RD) is a rare, chronic fibroinflammatory condition that can affect multiple organ systems. Cutaneous involvement in IgG4-RD is even rarer. Moreover, the clinical manifestations of primary IgG4-related skin disease (IgG4-RSD) are often atypical, making it easy for clinicians to misdiagnose or overlook the condition, leading to delays in diagnosis and treatment.

**Case presentation:** We report the case of a 36-year-old male who was admitted to the hospital due to recurrent papules and pustules at multiple sites across the body for 8 years. He was misdiagnosed with hidradenitis suppurativa at an outside hospital and received long-term anti-infective therapy and irregular prednisone treatment, but experienced recurrent episodes. This admission was prompted by worsening skin lesions complicated by sepsis. Based on clinical clues including chronic recurrent skin lesions, poor response to anti-infective therapy, and symptom relief with corticosteroids but frequent relapses, we performed targeted serum IgG4 testing and skin biopsy, ultimately confirming the diagnosis of primary IgG4-RSD. After treatment with a standardized regimen of glucocorticoids combined with azathioprine as a steroid-sparing agent, the skin lesions significantly improved. At the 2-month follow-up after discharge, no new skin lesions developed, pre-existing lesions had largely healed, and serum IgG4 levels returned to normal.

**Conclusion:**

Primary IgG4-RSD is rare and diagnostically challenging, as it can mimic a wide range of diseases and its pathogenesis remains incompletely understood. A definitive diagnosis requires markedly elevated serum IgG levels, characteristic histopathological findings, and rigorous exclusion of mimicking disorders. Additionally, the condition tends to relapse during treatment. Therefore, enhancing clinicians’ understanding of IgG4-RD is crucial.

## Introduction

Immunoglobulin G4-related disease (IgG4-RD) is a chronic immune-mediated fibroinflammatory disorder (1). Its pathogenesis is multifactorial, involving genetic and environmental factors, as well as innate and adaptive immunity, autoantigens and autoantibodies, and interactions between T cells and B cells. Elevated serum IgG4 levels, along with pathological features such as lymphoplasmacytic infiltration rich in IgG4-positive plasma cells, storiform fibrosis, obliterative phlebitis, and eosinophilic infiltration, are characteristic findings. The disease can affect nearly any organ, with multi-organ involvement being common (2). Primary IgG4-related skin disease (IgG4-RSD) is a subtype in which only the skin is involved, without other organ involvement. Cutaneous involvement is rare in IgG4-RD, and isolated cutaneous involvement in IgG4-RSD is even rarer. Due to the diversity of organs involved and the poor specificity of signs and symptoms, this disease is often underdiagnosed or misdiagnosed. The diagnosis is based on a comprehensive analysis of clinical features, serum IgG4 levels, imaging, and histopathology (3, 4). In IgG4-RSD, especially in early or skin-limited cases, storiform fibrosis and obliterative phlebitis are not required for diagnosis. As confirmed in the literature (5), cutaneous IgG4-RSD may present only with dense lymphoplasmacytic infiltration and an elevated IgG4/IgG ratio, without fibrosis or phlebitis. It is worth noting that elevated serum IgG4 or infiltration of IgG4-positive plasma cells in tissues alone is insufficient for diagnosis, and other diseases must be excluded (6). Therefore, early multidisciplinary diagnosis and management, combined with the active use of ancillary tests, are crucial for reducing misdiagnosis and improving diagnostic accuracy. Corticosteroids are the first-line treatment for this condition (7, 8). This case, presenting with papules, pustules, and abscesses as the sole manifestations and misdiagnosed for eight years, represents a highly atypical form of primary IgG4-RSD. Reporting this case aims to enhance awareness of insidious phenotypes, emphasize the critical value of skin biopsy and serum IgG4 testing, provide a replicable diagnostic approach for clinical practice, and offers clear novelty and educational value.

## Case description

**Figure 1:**
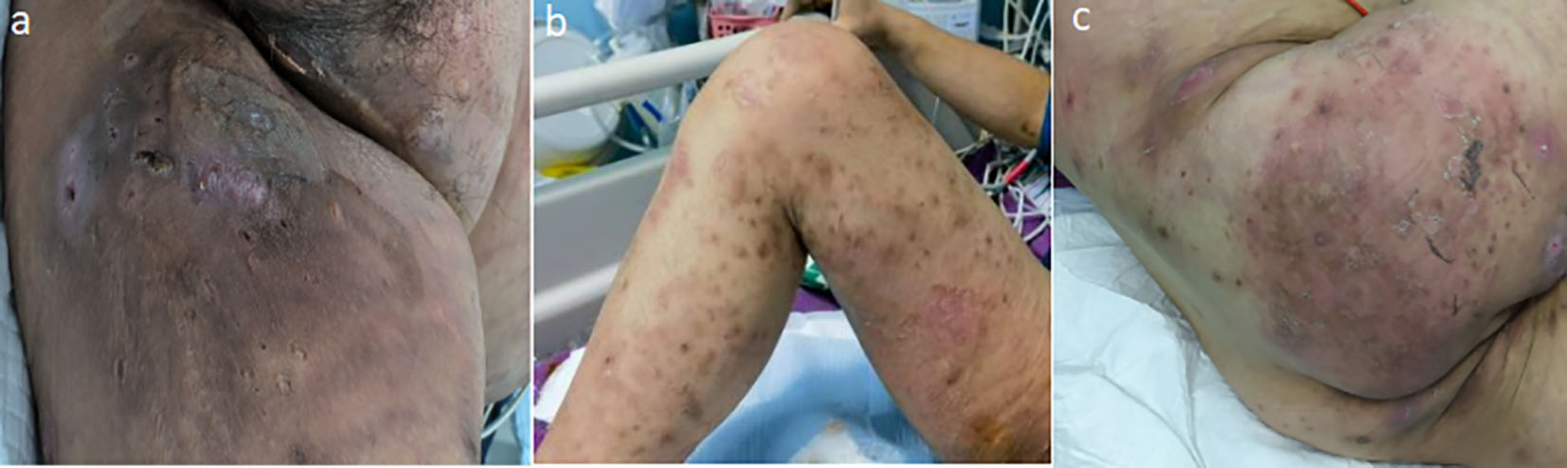
Clinical manifestations at admission. **(a)** Multiple dark red nodules and abscesses in the groin with ulceration, crusting, and skin thickening; **(b)** Multiple scattered red papules and pustules on the right lower extremity; **(c)** Large confluent infiltrative erythema and plaques on the right buttock with ulceration, desquamation, and scarring.

**Figure 2:**
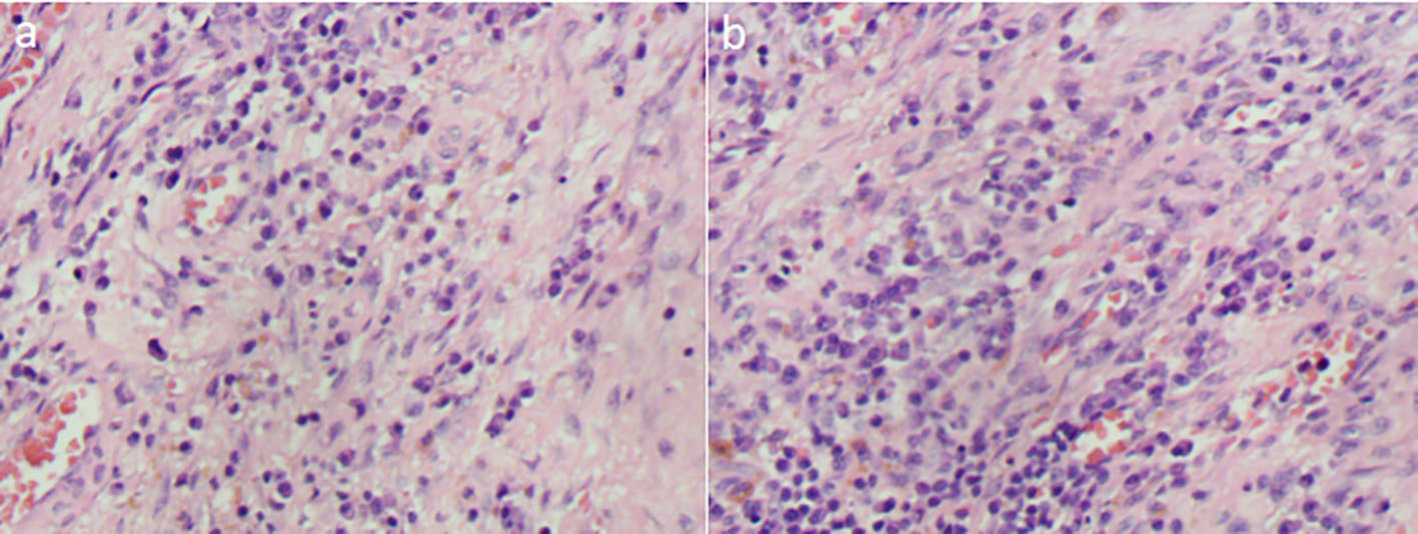
Histopathology of skin biopsy (H&E staining). **(a, b)** Massive plasma cell infiltration with scattered eosinophils.

**Figure 3:**
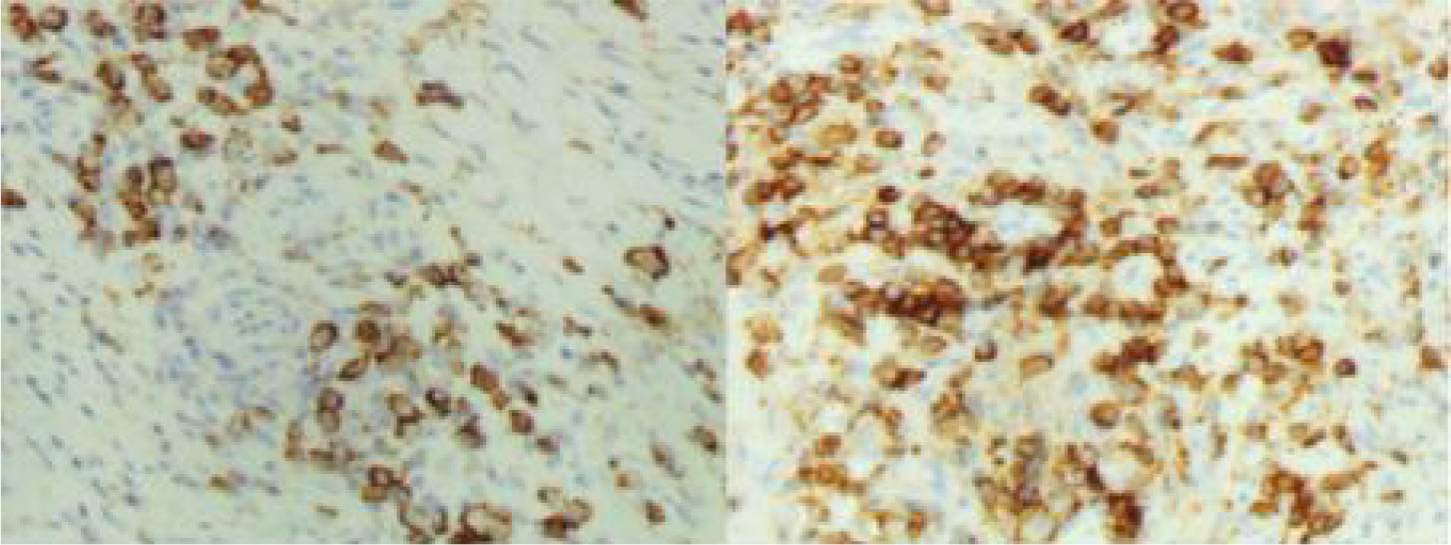
Immunohistochemical staining. Abundant IgG4-positive cells, with an IgG4-positive plasma cell/IgG-positive plasma cell ratio >40%.

**Figure 4:**
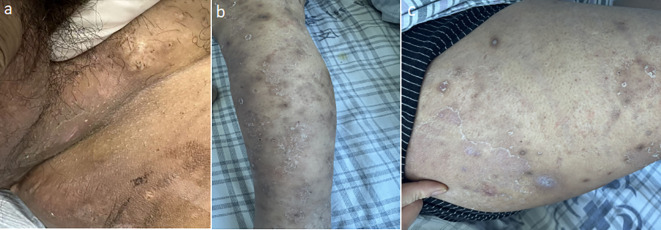
Clinical manifestations after 2 months of treatment. **(a)** Inguinal region; **(b)** Right lower limb; **(c)** Right buttock. The original large confluent erythema and inflammatory infiltration had largely resolved, with only residual scattered dark red macules and patches remaining.

A 36-year-old male presented with recurrent papules and pustules on the face, perineum, and both lower limbs for over 8 years, with worsening accompanied by pain for 10 days prior to admission. He had a 2-year history of hypertension, for which he took regular medication with good blood pressure control, and a 6-month history of type 2 diabetes mellitus, for which he did not take regular medication or monitor his blood glucose. There was no history of drug or food allergies, and no similar symptoms were reported among his parents, siblings, or children. The patient had a history of recurrent papules and pustules on the perineum and both lower limbs, for which he received irregular treatment at clinics with recurrent episodes. He was previously diagnosed with hidradenitis suppurativa at an outside hospital and received long-term anti-infective therapy and irregular prednisone treatment, but the condition continued to recur. This time, the patient’s skin lesions had increased in extent, spreading to both shoulders and the face, leading him to seek care at our hospital. On admission, physical examination revealed scattered multiple red papules and pustules in the groin, right lower limb, and right buttock, some coalescing into plaques with local ulceration and exudation (**Figure 1**). Using a ruler-based measurement method, the total area of involved skin was approximately 100 cm², of which the area of active erosion and exudation was approximately 20 cm², mainly located in the groin region. According to the skin domain score of the IgG4-RD Responder Index (IgG4-RD RI) developed by Carruthers et al. (9) in 2012, the patient had extensive skin lesions with ulceration and sepsis at admission, scoring 4 points (emergency situation, score ×2 = 8 points). Since the onset of the disease, the patient had no symptoms such as dry eyes or dry mouth. The patient had a disease course lasting 8 years, characterized by recurrent skin lesions, poor response to conventional anti-infective therapy, temporary relief with systemic corticosteroids but frequent relapses, which is inconsistent with the typical course of infectious skin diseases. The skin lesions primarily consisted of chronic infiltrative plaques, nodules, and abscesses, with scar formation and skin thickening after repeated episodes. Based on these features, we strongly suspected an immune-mediated disease and therefore included IgG4-RSD in the differential diagnosis. Targeted serum IgG4, IgG subclasses, and skin biopsy were subsequently performed. The patient had not undergone a skin biopsy for 8 years, mainly because outside hospitals had treated the condition as hidradenitis suppurativa or other infectious diseases without considering immune-mediated skin diseases, leading to a delay in diagnosis. After admission, a comprehensive autoimmune antibody panel (including anti-PM-Scl antibody, anti-mitochondrial M2 antibody, anti-ribosomal P protein antibody, anti-histone antibody, anti-nucleosome antibody, anti-cyclin antibody, anti-centromere protein B antibody, anti-Jo-1 antibody, anti-Scl-70 antibody, anti-SSB antibody, anti-recombinant RO-52 antibody, anti-SSA antibody, anti-Sm antibody, anti-U1RNP antibody, anti-double-stranded DNA antibody, anti-cyclic citrullinated peptide antibody, rheumatoid factor, and antinuclear antibody [ANA]) showed no abnormalities. Infectious screening (HIV, hepatitis, syphilis, toxoplasma, rubella virus, cytomegalovirus, herpes simplex virus) was negative. Blood tests revealed white blood cell count 12.5×10^9^/L, neutrophil percentage 82.3%, CRP 68.5 mg/L, PCT 0.87 ng/mL, indicating secondary bacterial sepsis due to extensive cutaneous ulceration, rather than being intrinsic to IgG4-RD itself. Humoral immunity tests showed normal IgA and IgM, IgG 44.10 g/L, total IgE 427 IU/mL, and IgG4 26.71 g/L(reference range: 0.03–2.01 g/L), which was 13-fold above the upper limit of normal. Head and pelvic CT showed no diffuse or focal enlargement of the pancreas, salivary glands, lacrimal glands, biliary tract, or retroperitoneum. Cervical lymph node ultrasound demonstrated multiple enlarged bilateral cervical lymph nodes (largest 1.5 cm×0.6 cm) with regular morphology, clear corticomedullary differentiation, and intact hilum, consistent with reactive hyperplasia. A full-thickness excisional skin biopsy was performed at the most severely affected site in the groin. Histopathology revealed a dense infiltration of lymphocytes and plasma cells (**Figure 2**). Immunohistochemistry showed Ki-67 (+, ~20%), CD34 (interstitial dot-like +), IgG4 (+, >10 cells/HPF), CD38 (+), SMA (interstitial focal +), and CD138 (+). Special stains (PAS, GMS) were negative. The IgG4+/IgG+ plasma cell ratio exceeded 40% (**Figure 3**). According to the 2020 revised Japanese diagnostic criteria for IgG4-RD (10), this patient met the following: clinical examination revealed localized swelling of the skin; serological testing showed elevated serum IgG4 levels; and most importantly, histological examination demonstrated diffuse plasma cell infiltration, with a ratio of IgG4+ plasma cells to total plasma cells >40% and >10 IgG4+ plasma cells per high-power field. The patient was definitively diagnosed with IgG4-RSD. The patient was treated with intravenous methylprednisolone sodium succinate 40 mg/day for 7 days. After the skin lesions and sepsis were controlled, the treatment was switched to oral prednisone 30 mg/day as sequential therapy. Once the condition stabilized, the dose was reduced by 5 mg every 1–2 weeks. Azathioprine at 2.5 mg/kg was added as a steroid-sparing agent to facilitate tapering of corticosteroids and reduce the risk of relapse. Prednisone was continued until it was tapered to a maintenance dose of 10 mg/day. The total planned duration of corticosteroid therapy was 6 months. The patient had comorbid type 2 diabetes mellitus and hypertension. During treatment, fasting and 2-hour postprandial blood glucose levels were monitored daily, and basal-bolus insulin therapy was administered to regulate blood glucose. Blood pressure was monitored twice daily, and the original antihypertensive regimen was maintained with minor adjustments to ensure stable blood glucose and blood pressure control. Simultaneously, bacterial culture and antimicrobial susceptibility testing of purulent discharge were performed. Sensitive antibiotics were administered promptly along with the initial methylprednisolone therapy for appropriate anti-infection treatment. Antibiotics were discontinued promptly once the infection was controlled and clinical and laboratory findings indicated resolution of infection. After one week of treatment, follow-up blood tests showed: white blood cell count 7.8×10^9^/L, neutrophil percentage 65.2%, CRP 12.3 mg/L, PCT 0.15 ng/mL, indicating a significant decrease in inflammatory markers. At the 2-month follow-up after discharge, the patient had no new pustules or abscesses in the perineal area or lower limbs, and the original skin lesions had largely healed (**Figure 4**). The IgG4-RD RI skin score was 0 (complete remission), and serum IgG4 levels returned to normal. During the 6-month follow-up period, the patient experienced no systemic symptoms such as dry mouth, dry eyes, abdominal pain, or jaundice, and follow-up imaging revealed no new abnormalities.

## Discussion

IgG4-RD is a chronic immune-mediated fibroinflammatory disease (11) that can present with a variety of skin manifestations, collectively referred to as IgG4-RSD (12). IgG4-RSD is relatively uncommon and is more frequently observed in middle-aged and elderly males. Clinically, erythematous papules, plaques, and nodules are the most common morphologies, and may be accompanied by pruritus (13). However, skin-limited manifestations have been documented as the initial presentation of IgG4-RD and may precede the development of systemic manifestations by many years (14–16), making it prone to misdiagnosis as an infectious skin disease. The patient in this case presented with recurrent papules and pustules as the main manifestation and was misdiagnosed with hidradenitis suppurativa for 8 years, indicating that this subtype has highly atypical clinical features. The diagnosis of IgG4-RD can be challenging, especially when clinical presentations are unusual or histological features are atypical. Many other diseases (both neoplastic and non-neoplastic) can mimic IgG4-RD on clinical or histological examination (17). Maintaining a high level of clinical vigilance is crucial to avoid diagnostic delays. IgG4-RSD can overlap clinically and pathologically with various skin diseases, and differentiation from the following conditions is particularly necessary (**Table 1**): (1) Hidradenitis suppurativa: Predilection for the groin and axillae, presenting with recurrent abscesses, sinus tracts, and scarring; pathology predominantly shows neutrophilic infiltration and destruction of sweat gland ducts, without an increase in IgG4-positive plasma cells; serum IgG4 is normal; anti-infective treatment is effective. In this case, there was no significant neutrophilic abscess, IgG4 was markedly elevated (18), and conventional anti-infective therapy was ineffective, allowing exclusion. (2) Chronic infectious skin diseases: Deep fungal infections and non-tuberculous mycobacterial infections may cause reactive elevation of IgG4, but etiological examination is positive, pathogens are visible on pathology, and standardized anti-infective therapy is effective. In this case, despite control of secondary infection, skin lesions continued to recur for 8 years; serum IgG4 was markedly elevated; no pathogen was found on pathology; and there was a significant response to corticosteroids, allowing exclusion. (3) Sarcoidosis, plasmacytosis, chronic inflammation, etc.: These may show lymphoplasmacytic infiltration and mildly elevated IgG4, but lack typical storiform fibrosis; the IgG4+/IgG+ ratio is usually <40%, and they can be excluded by combining clinical and pathological findings (6, 19). It is particularly important to emphasize that elevated serum IgG4 and an elevated tissue IgG4+/IgG+ ratio are only supportive indicators and cannot be used alone for diagnosis. The diagnosis of IgG4-RD must be made by combining clinical manifestations, pathological features, and the exclusion of other diseases. A clear distinction should be made between diagnostic criteria and classification criteria: The 2019 ACR/EULAR criteria (4) are classification criteria for IgG4-RD, primarily used for patient enrollment and categorization in clinical research, and they cannot replace comprehensive clinical diagnosis. The diagnosis in this case was based on clinical manifestations, serology, histopathology, and exclusion of other diseases, representing a clinical diagnosis rather than a simple application of classification criteria. According to the classification of IgG4-related skin diseases proposed by Tokura et al. (5), skin lesions can be divided into primary and secondary types. In this case, skin biopsy showed dense infiltration of IgG4-positive plasma cells, and imaging and long-term follow-up excluded involvement of other organs, consistent with the diagnosis of IgG4-RSD. This case, characterized by recurrent papules and pustules with strong subtlety and clinical difficulty in recognition, along with lack of a definitive diagnosis and standardized treatment in the early stage, led to disease prolongation. Subsequent infection further increased diagnostic and therapeutic difficulty. When skin lesions serve as the initial manifestation of systemic IgG4-RD, timely recognition of this rare presentation is crucial, because delayed diagnosis and treatment may lead to organ fibrosis and irreversible damage (20, 21). Compared with most visceral organ biopsies, which are difficult to obtain, skin biopsy is simple and minimally invasive (22). For skin lesions of unknown etiology that respond poorly to topical corticosteroids, IgG4-RSD should be included in the differential diagnosis, and skin biopsy and serum IgG4 testing should be recommended. Corticosteroids are first-line therapy for IgG4-RD, but relapse is common during tapering (23). Immunosuppressants can reduce corticosteroid dosage and prevent relapse (24–26). Conventional synthetic disease-modifying antirheumatic drugs (csDMARDs) such as mycophenolate mofetil and azathioprine can be used as steroid-sparing agents (27, 28); rituximab may be used in refractory or relapsed cases (23, 29). In this case, due to the long disease duration and recurrent relapses, azathioprine was added early to achieve smooth corticosteroid tapering and sustained remission.

**Table 1:** Key differential diagnoses of IgG4-RSD in this patient.

Diagnosis	Clinical features	Histopathological features	Serum IgG4	Response to treatment
IgG4-related skin disease (this case)	Chronic recurrent papules, plaques, abscesses; poor response to antibiotics; frequent relapses	Dense lymphoplasmacytic infiltration; IgG4+ plasma cells >10/HPF; IgG4+/IgG+ plasma cell ratio >40%	Markedly elevated	Excellent response to glucocorticoids and immunosuppressants
Hidradenitis suppurativa	Recurrent abscesses, sinus tracts, scars in intertriginous areas	Neutrophilic infiltration; destruction of sweat gland ducts; no increased IgG4+ plasma cells	Normal	Effective to antibiotics
Hidradenitis suppurativa	Recurrent abscesses, sinus tracts, scars in intertriginous areas	Neutrophilic infiltration; destruction of sweat gland ducts; no increased IgG4+ plasma cells	Normal	Effective to antibiotics
Chronic infectious dermatoses	Positive pathogen identification; obvious infectious etiology	Microabscesses; detectable pathogens; no typical storiform fibrosis	Mildly elevated in reactive conditions	Effective to standard anti-infective therapy
Sarcoidosis/Plasma cell hyperplasia/Other chronic inflammatory disorders	Non-infectious granulomas or plasmacytosis; no typical abscesses	Lymphoplasmacytic infiltration; no typical storiform fibrosis; IgG4+/IgG+ ratio usually <40%	Normal or mildly elevated	Variable response to glucocorticoids

The etiology of IgG4-RD is currently unclear; genetic susceptibility and immune dysregulation may be important factors. This disease can mimic various conditions, including malignancies, infections, and autoimmune diseases. Cutaneous involvement is relatively rare, making early diagnosis more difficult (30, 31). In summary, this case represents early typical IgG4-RD presenting primarily with non-specific skin lesions. It reminds clinicians that when encountering recurrent papules, eczema, psoriasis-like, or pustular lesions that respond poorly to topical corticosteroids or anti-infective treatment, a comprehensive evaluation and early multidisciplinary consultation should be pursued to establish a diagnosis. IgG4-RD with skin as the first and only manifestation is rare, and its main features lack specificity, resembling other inflammatory skin diseases. Therefore, this case can serve as a reference for clinicians. Further accumulation of more cases of IgG4-RSD skin lesions is needed to systematically evaluate whether these diagnostic tools can provide disease-specific clues, ultimately offering new perspectives for the diagnosis of this disease.

## Data Availability

The original contributions presented in the study are included in the article/supplementary material. Further inquiries can be directed to the corresponding authors.
